# Intravenous iron infusion as an alternative to minimize blood transfusion in peri-operative patients

**DOI:** 10.1038/s41598-020-75535-2

**Published:** 2020-10-27

**Authors:** Alin Ionescu, Abhinav Sharma, Nilima Rajpal Kundnani, Alexandra Mihăilescu, Vlad Laurențiu David, Ovidiu Bedreag, Dorel Săndesc, Anca Raluca Dinu, Mihai Alexandru Săndesc, Nicolae Albulescu, Răzvan Gabriel Drăgoi

**Affiliations:** 1 Department of Family Medicine, “Victor Babes” University of Medicine and Pharmacy, Timisoara, Romania; 2 Department of Cardio-Vascular Rehabilitation & Family Medicine, “Victor Babes” University of Medicine and Pharmacy, Timisoara, Romania; 3 Department of Physiology, “Victor Babes” University of Medicine and Pharmacy, Timisoara, Romania; 4 Department of Genetics, “Victor Babes” University of Medicine and Pharmacy, Timisoara, Romania; 5 Department of Pediatric Surgery and Orthopedics, “Victor Babes” University of Medicine and Pharmacy, Timisoara, Romania; 6 Department of Anesthesia and Intensive Care, “Victor Babes” University of Medicine and Pharmacy, Timisoara, Romania; 7 Department of Physical Medicine and Balneology, “Victor Babes” University of Medicine and Pharmacy, Timisoara, Romania; 8 Department of Orthopedics and Traumatology, “Victor Babes” University of Medicine and Pharmacy, Timisoara, Romania; 9 “Victor Babes” University of Medicine and Pharmacy, Timisoara, Romania; 10 Department of Rehabilitation Medicine, “Victor Babes” University of Medicine and Pharmacy, Timisoara, Romania

**Keywords:** Health care, Medical research

## Abstract

Despite the reported benefits of intravenous iron therapy (IVIT) for correcting iron deficiency anemia (IDA) before any major surgery and the evidence thereof, perioperative allogenic blood transfusion (ABT) practice is still considered as the only viable option by some clinicians worldwide. As ABT increases the likelihood of infections, cardiac complications, longer hospital stays and mortality among the patients, the practice of ABT should only be reserved for critical cases (Hb level < 7 g/dl). Timely iron studies and iron replenishment (oral/IV) of prospective surgical patients could help decrease the ABT practice, and prove beneficial from both the clinical and economic standpoint. Evidence based patient blood management guidelines should be developed and standardized for use by clinicians worldwide. These guidelines should include specific instructions on timely assessment of surgical patients for correction of their IDA by either oral iron supplementation, if time permits, or by using IVIT such as ferric carboxymaltose (FCM) in emergency surgeries and in patients with functional ID. This study was conducted to explore the clinical benefits of the timely administration of IV-FCM in iron-deficient preoperative patients during 2017–2018 and compare the results thereof with that of the ABT. Based on the IDA treatment plan of 2953 patients, 11.14% cases were administered IV FCM (Group 1), 11.58% cases received ABT (Group 2), while the remaining 77.27% of anemic cases received neither ABT nor IV FCM (Group 3). The results indicate that the IV FCM administration reduces the need for ABT and thus minimizes its associated side effects. The findings of our study concur with the favorable outcomes reported by the other similar studies.

## Introduction

Anemia is a global health problem affecting a quarter of the world’s population. Iron deficiency (ID) is considered to be the leading cause in both men and women, albeit women are more commonly affected^1–3^. The ID that leads to anemia could be of absolute or functional deficiency in nature. The absolute deficiency occurs due to the mismatch between iron uptake and utilization^1^ and functional deficiency occurs due to the inflammation associated impaired iron release from enterocytes, macrophages and or hepatocytes^1,4,5^. Unlike the absolute ID, functional ID develops in spite of adequate iron stores as hepcidin overexpression in inflammation blocks iron’s release from hepatocytes and macrophages and also its absorption by enterocytes, forming the basis of anemia of chronic disease (ACD)^1,4,5^. Therefore, the functional and or the malabsorption syndromes’ related deficiency and the limited rate of intestinal absorption of oral iron and the consequential sluggish replacement warrants the use of IV iron such as ferric carboxymaltose (FCM)^1^. Also, poor patient compliance due to side effects limits the efficacy of oral iron^1^. However, being inexpensive and readily available, oral iron is still a viable option to start replacement in patients with mild anemia (Hb, 11.0–12.9 g/dl in men and 11.0–11.9 g/dl in non-pregnant women), but it should be continued only if the response to therapy is optimal (Hb increase by 2 g/dl within 4–8 weeks) and well tolerated^1^. In contrast, IV iron leads to a faster replacement^1,6–8^, and diminishes the rate of recurrence of iron deficiency anemia in the long term^1,9,10^. The use of allogenic blood transfusion (ABT) as an alternative means to manage ID may be considered for treating critical anemia (Hb level < 7 g/dl), acute myocardial ischemia, hemodynamically unstable active bleeders, or in the case of treatment failure with the other options^1,11–13^.

Factors that are responsible for causing iron depletion are blood loss (most common), poor nutrition, age, pregnancy, socioeconomic status and or various pathological conditions^14^. Additionally, many individuals may develop ID in response to therapies such as nonsteroidal anti-inflammatory medications^14^. It has been reported that in certain subgroups such as patients who have colorectal cancer or heavy menstrual bleeding, the occurrence of preoperative anemia is as high as 57%^14–16^ and in the non-cardiac surgery patients it is estimated to be around 39%^14,17^. Regardless of whether the ID or iron deficiency anemia (IDA) develops due to a disease process and or therapy, it is a cause of concern in patients who undergo surgery because it adversely impacts the prognosis^14^.

Along with preoperative anemia, ABT and significant perioperative blood loss have also been associated with negative clinical outcomes^14,18^. Administration of allogenic blood to perioperative anemic patients is associated with increased rates of infections, cardiac complications, prolonged hospital stays and more deaths^14,17,19–21^. Nonetheless, despite the existing evidence of how even small amounts of transfused allogenic blood impacts morbidity and mortality negatively, anemia correction by ABT is still quite prevalent^14,22^. Although patient blood management (PBM) programs have been successfully developed and implemented in some countries^14,23,24^, most countries still lack them and thus persisting evidence gaps and skepticism give rise to varying ABT practices among the clinicians^14,25^. One of the objectives of PBM is correction of anemia before surgery, especially before major surgical procedures^14,18,25,26^. ID assessment should ideally be done 4 weeks prior to a major surgery to allow correction of reversible hemopoietic deficiencies^14,26–28^. For the correction of ID or IDA, both IV and oral iron are considered effective^14,29,30^, but only oral iron therapy has managed to become standard practice^14,30,31^. However, existing evidence suggests that the treatment with IV iron in elective surgeries and urgent cases benefits patients and minimizes ABT and its associated adverse events^14,26,32^. The mounting evidence supporting the predicted positive effect of IV iron on iron levels in the immediate postoperative period and subsequent weeks after hospital discharge would encourage the widespread use of IV iron (e.g. FCM) in preoperative IDA patients.

## Methods

The objective of the study was to conduct retrospective analyses of the medical records of preoperative IDA patients who were hospitalized for surgeries between Jan 2017 and Dec 2018. For conducting the audit, we obtained the data from Department of Public Health, Management and Improvement in Healthcare, Bucharest (SNSPDPMS) and National Program database. We extracted and analyzed the data of 10,571 surgical patients based on the following criteria: (a) age > 18 with anemia, (b) Hb < 12.0 g/dl for women and Hb < 13.0 g/dl for men, (c) ferritin < 300mcg/l, (d) transferrin saturation < 25%, (e) pre-op time of 1–20 days. Out of 10,571 patients, 2953 patients had anemia and were divided into 3 groups to compare the data pertinent to the perioperative management of anemia. As a part of the perioperative care, group 1 received IV FCM (available as 10 ml/500 mg vials and a maximum dose of 20 ml/1000 mg was administered per week), group 2 received ABT and group 3 received neither IV FCM nor ABT. Our main objective was to report the clinical outcomes of the preoperative IDA patients who received perioperative IV FCM. We also explored the presence of hypophosphatemia that may have occurred as a side effect of IV FCM administration.

### Ethics approval and informed consent statement

Prior to the commencement of the study, ethics approval and written consent was obtained from all the relevant persons or authorities. This manuscript has not been published and or submitted for consideration for publication elsewhere. Approved by all of our authors who have no conflicts of interest to disclose, I am submitting this manuscript to your journal.

The study was approved by the ‘Comisia Locala de Etica pentru cercetare Stiintifica a Spitalului Clinic Judetean de Urgenta’ Timisoara, in accordance to the law art 167 of legal code nr. 95/2006, art. 28, Cap VIII, of law 904/2006 and in accordance to EU GCP Directives 2005/28/EC, International Conference of Harmonisation of Technical Requirements for Registration of Pharmaceuticals for Human Use (ICH) and declaration of Helsinki—Recommendations Guiding Medical Doctor in Biomedical Research Involving Human Subjects. All the steps of the study were conducted in accordance with the above guidelines, conforming to the standard operational procedures for clinical studies approved for Sp Judetean, (Timis Emergency County Hospital, Romania). The details of which can be found on the official hospital website https://www.hosptm.ro/cercetare-dezvoltare/studii-clinice/.

This retrospective study was conducted in our University hospital and as a part of routine procedure informed written consent forms stating that the data can be used for future medical research purpose were signed by each patient at the time of admission in the hospital.

## Results

**Figure 1:**
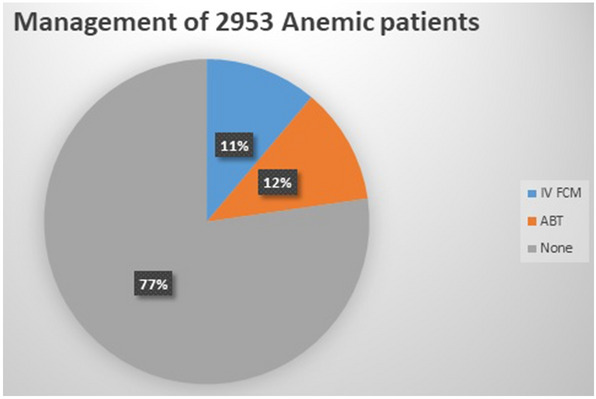
Management of 2953 anemic patients.

**Figure 2:**
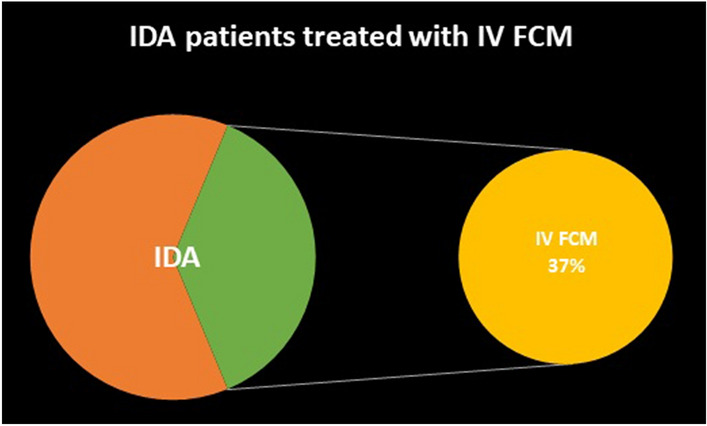
IDA patients treated with IV FCM.

The data analyses of the surgical records of 10,571 patients (5370 from 2017 and 5201 from 2018) who underwent surgery yielded the following results: 2953 of the 10,571 (27.93%) cases had anemia (Hb < 12.0 g/dl for women and Hb < 13.0 g/dl for men) (Fig. 1), out of which 879 (29.76%) patients had IDA. 329 of the 2953 (11.14%) cases were administered IV FCM (around 37% of total IDA cases) and were classified as Group 1 (Fig. 2), 342 of the 2953 (11.58%) cases received ABT and were referred to as Group 2, while the remaining 2282 (77.27%) of 2953 mildly anemic cases received neither ABT nor IV FCM and were designated as Group 3 (average Hb;11.95 g/dl in men and 11.45 g/dl in non-pregnant women).

The Group 1 patients received 511 doses of IV FCM with an average of 1.55 dose/patient (1000 mg IV infusion diluted in sterile 0.9% m/v sodium chloride over 15 min and or 500 mg as a slow IV bolus injection). No major side effects were reported following the IV FCM therapy. The analysis showed that the IV iron administration enhanced replacement of iron stores (serum ferritin 235 vs. 110 ng/ml) and a higher mean Hb concentration (12 vs 11 g/dl) 4 weeks post-op. However, no differences in the discharge Hb levels, morbidity, mortality, and quality of life were noted. 31 of 329 patients (9.42%) in group 1 had transient asymptomatic hypophosphatemia, no other symptoms were reported among the group 1 (FCM) patients.

## Discussion

The results of our study indicate the predominant reliance on ABT rather than IV iron in the perioperative management of IDA in Romania. Despite the reported transfusion-associated higher costs and risks, it is by far the default treatment for IDA in the perioperative period^14,33–35^. Our study demonstrates IV iron as a viable alternative to reduce ABT and its associated costs and risks in managing perioperative anemia. Though the Hb levels of the patients at discharge were observed to be the same between the groups, Hb levels in the 1st group (IV FCM group) were higher by 1 g/dl on an average at the follow-up visits, 4 weeks’ post-surgery. This benefit of perioperative administration of IV iron can be attributed to its ability to replenish iron stores required for erythropoiesis by the bone marrow, unlike ABT in which transfused RBCs are cleared from the circulation rapidly and have a shorter lifespan than normal RBCs^14,36^. Concurring with our finding, the significance of IV iron in reducing the proportion of transfused units of RBCs has been previously demonstrated in a clinical setting by Munoz^14,37^.

Functional IDA is a common feature of most major surgeries. Despite adequate iron stores in functional IDA, the unavailability of iron for erythropoiesis is due to the altered release of iron from macrophages and its subsequent incorporation into transferrin^38–42^. Results from various studies support the use of high dose IV iron infusion regimens to overcome this iron immobilization issue, wherein the iron saturated macrophages circumvent the hepcidin block by causing over-expression of ferroportin and the subsequent increase in transferrin saturation^43^. Therefore, preoperative IV iron administration can prevent postoperative anemia in patients with functional IDA^42,44^. Still, most often RBC transfusion is the strategy of choice to tackle perioperative IDA in the management of patients undergoing major surgeries, a finding that is also reported by Shin et al.^42^.

Supplementation with oral iron tends to be the first-line treatment for most IDA patients because of its low cost and easy availability. However, in conditions such as functional IDA and trauma or surgery, oral iron therapy is ineffective due to poor GI absorption and slow delivery and adverse effects, respectively. As there is rapid and direct binding of IV iron to plasma transferrin, the erythropoietic effect increases about 5 times and lasts for 7–10 days^42,45^. The underlying mechanism that has been stated is based on the data from in vitro studies, which showed that approximately 45 mg of iron can be maintained in the plasma after the administration of 1000 mg IV iron, wherefrom the small proportion (4–5 mg) and the large proportion is taken up by transferrin and macrophages, respectively^43^. Unlike many others studies which have highlighted the IV iron associated positive outcomes as compared with the oral iron therapy, our team’s primary focus was to explore the viability of IV iron therapy (IVIT) as an alternative to ABT, if and when possible, in managing perioperative ID or IDA and decreasing the rate of ABT.

IV iron formulations comprise an iron core and carbohydrate shell, the shell which steadies their release in the circulation^42^. Among the most commonly, reported adverse effects of IV iron are headache, vomiting, chest tightness, arthralgia, fever and hot flushes^42,46^. However, more serious adverse effects such as anaphylaxis, infection, oxidative stress, hypophosphatemia, hypotension, and even mortality have also been reported^42,47^. For instance, the use of high molecular weight iron dextran (Dexferrum) was discontinued due to its potential for causing anaphylaxis^42^. In contrast, the large systematic review of IVIT safety conducted by Avni et al., which included 103 randomized control trials and 10,390 patients^42,48^ reported no association of IVIT with an increased risk of neurologic, respiratory, and cardiovascular adverse effects^42^. Also, no increase in mortality and adverse effects which required discontinuation were reported^42^. However, the common occurrence of IV iron associated infusion site reactions were reported^42,48^.

Considering the safety profile (low immunogenic potential) and the single total dose infusion advantage of IV FCM^43^, plenty of data suggest the use of IV FCM for preoperative ID or IDA management, and therefore our county hospital also prefers its administration, if and when required. FCM has a carboxymaltose outer shell, the shell which helps in sustained iron release and its optimal delivery to the tissues^32,42,49^. At our hospital, IV FCM is usually administered as a single infusion over 15 min, to a maximum weekly dose of up to 1000 mg (European Union recommendation). IV FCM is sufficient for replacing iron stores during the perioperative period^42^ and the building evidence suggests that it may help minimize the need for ABTs during the perioperative period. According to the most recent guidelines^24,42,50^, the preoperative patients with functional IDA who are at risk of excessive blood loss during surgery are recommended to get IVIT to improve outcomes^42^. Managing preoperative IDA that affects 30% of patients^51^ with the use of IV FCM can improve clinical outcomes. The administration of IV FCM can substantially reduce the need for blood products in the management of preoperative anemia^24,51,52^ and reduce the length of hospital stay^14,51^. Our study indicates the clinical benefits of IV FCM administration over the traditional ABT in the management of preoperative IDA, the findings which were also reported by Froessler et al.^51^.

## Conclusion

Our results concur with the previous observational and experimental studies on the subject and thus contribute to the growing body of evidence in favor of perioperative IV iron (e.g. FCM) use in IDA patients undergoing major surgeries with expected high blood loss. However, the treatment with IV iron is not yet covered by many national health insurance programs, a factor that may also be limiting IV iron’s widespread use in the perioperative management of IDA patients. Therefore, studies are required to explore and highlight the economic benefits of IV iron in contrast to ABT and convince the regulatory authorities for the inclusion of IV iron (e.g. FCM) on the national insurance programs. Furthermore, implementing relevant protocols will also reduce transfusion related risks in IDA patients undergoing major surgeries.
